# FGFR1 inhibits skeletal muscle atrophy associated with hindlimb suspension

**DOI:** 10.1186/1471-2474-8-32

**Published:** 2007-04-10

**Authors:** John Eash, Aaron Olsen, Gert Breur, Dave Gerrard, Kevin Hannon

**Affiliations:** 1 Department of Basic Medical Sciences, Purdue University, West Lafayette, Indiana 47907, USA; 2 Department of Animal Sciences, Purdue University, West Lafayette, Indiana 47907, USA; 3 Department of Veterinary Clinical Sciences, Purdue University, West Lafayette, Indiana 47907, USA

## Abstract

**Background:**

Skeletal muscle atrophy can occur under many different conditions, including prolonged disuse or immobilization, cachexia, cushingoid conditions, secondary to surgery, or with advanced age. The mechanisms by which unloading of muscle is sensed and translated into signals controlling tissue reduction remains a major question in the field of musculoskeletal research. While the fibroblast growth factors (FGFs) and their receptors are synthesized by, and intimately involved in, embryonic skeletal muscle growth and repair, their role maintaining adult muscle status has not been examined.

**Methods:**

We examined the effects of ectopic expression of FGFR1 during disuse-mediated skeletal muscle atrophy, utilizing hindlimb suspension and DNA electroporation in mice.

**Results:**

We found skeletal muscle FGF4 and FGFR1 mRNA expression to be modified by hind limb suspension,. In addition, we found FGFR1 protein localized in muscle fibers within atrophying mouse muscle which appeared to be resistant to atrophy. Electroporation and ectopic expression of FGFR1 significantly inhibited the decrease in muscle fiber area within skeletal muscles of mice undergoing suspension induced muscle atrophy. Ectopic FGFR1 expression in muscle also significantly stimulated protein synthesis in muscle fibers, and increased protein degradation in weight bearing muscle fibers.

**Conclusion:**

These results support the theory that FGF signaling can play a role in regulation of postnatal skeletal muscle maintenance, and could offer potentially novel and efficient therapeutic options for attenuating muscle atrophy during aging, illness and spaceflight.

## Background

Skeletal muscle atrophy can occur under many different conditions, including prolonged disuse or immobilization, cachexia, cushingoid conditions, secondary to surgery, or with advanced age. Atrophy is characterized by decreases in muscle mass, muscle mass to body ratio [1,2], and myofiber cross-sectional area [3]. Additional changes noted in atrophied skeletal muscle are a decrease in overall nucleic acid quantities, as well as a loss of myonuclei [4]. These physical characteristics are accompanied by decreased muscle strength [5], and increased propensity to muscle fatigue [6]. If severe enough, these changes can significantly impact the quality of life and productivity of an individual.

The mechanisms by which unloading of muscle is sensed and translated into signals controlling tissue reduction remains a major question in the field of musculoskeletal research. Two ubiquitin ligases have been identified that are involved with initiating approximately 50% of skeletal muscle atrophy associated with denervation [7]. However, there are currently no universally effective treatments available for skeletal muscle atrophy during disuse. Several strategies have been used in an attempt to attenuate or reverse muscle atrophy associated with disuse. The success of these attempts appears to depend on the action of growth factors used, the delivery system employed, and the model of atrophy studied. For example, intra-muscular injection of IGF-I protein inhibited muscle atrophy associated with denervation [8] and electroporation of IGF-I expression plasmid inhibited muscle loss associated with hind limb suspension [9], while administration of GH and/or IGF-I did not attenuate muscle atrophy associated with hind limb suspension [10]. Exercise was required in addition to administration of GH and IGF-I in order to inhibit muscle degeneration associated with disuse atrophy [10]. However, in certain clinical situations such as bed-ridden or casted patients, exercise is not an option when attempting to attenuate muscle atrophy.

The fibroblast growth factors (FGFs) have been shown to be intimately involved in fetal skeletal muscle growth and development and development of cultured skeletal muscle in vitro. FGFs are strong regulators of myoblast proliferation *in vitro *[11-17]. FGF expression and synthesis is localized to developing skeletal muscle masses *in vivo*, and in muscle cultures *in vitro *[18-27]. In these masses, FGF and FGFR expression is associated with the proliferative myoblast state [28]. *In vitro*, FGFR1, FGF1, FGF2, FGF6 and FGF7 are expressed in proliferating cultures of MM14 skeletal muscle precursor cells, and down-regulated upon differentiation [29]. Because of these expression patterns and regulatory effects mentioned above in skeletal muscle *in vitro*, it was believed classically that FGFs were responsible for stimulating proliferation of myogenic precursor cells *in vivo*. While this may be true for some FGFs, it is becoming clearer that not all FGF-family members will have a proliferation stimulating/differentiation inhibiting function with regard to skeletal muscle development *in vivo*. FGF5 and FGF4 significantly inhibit the development of differentiated skeletal muscle myofibers, without any significant stimulatory effect on myoblast proliferation *in vivo *[28,30].

The role of the FGF-family in regulation of postnatal skeletal muscle growth and maintenance is less clear. Expression of FGF-1 and 2 is significantly increased in the muscle fibers of facioscapulohumeral muscular dystrophy (FSHD) [31]. Electrically stimulated rat muscles showed a threefold increase of the mRNA levels of both FGF-1 and FGF-2 [32], indicating release during exercise and possible roles in the response of skeletal muscle to work. In addition to the changes seen in expression under various physiological conditions, FGF-2 can aid in muscle healing in vivo. Application of exogenous FGF-2 to injured muscle increased the number of regenerating fibers and the twitch and tetanus strength of muscle as compared to control muscles [33]. Injection of FGF-2 into the muscle of *mdx *mice increases the number of regenerating myofibers and this effect is positively correlated to the quantity of FGF-2 injected [34]. Physical trauma of skeletal muscle in FGF-6 null mice results in an inhibition of the healing process, and extensive fibrosis and myotube degeneration. When *mdx *mice were crossed with FGF-6 null mice the resultant offspring showed severe myopathies. These included myotube necrosis and large amounts of fibrosis, indicating inhibition of muscle regeneration [35].

The FGFs exert their biological effects on cells via both high affinity tyrosine kinase (FGFRs) and low affinity heparan sulfate proteoglycan (HSPGs) cell surface receptors [36]. In adult skeletal muscle, the two main FGFRs expressed are FGFR1 and FGFR4 [36]. FGFR1 is proposed to be a receptor for several members of the FGF family, and is believed to be associated with cellular proliferation. In electrically stimulated muscle, expression of FGFR1 was isolated to skeletal muscle fibers, and is doubled over non-stimulated muscles [32]. FGFR-4 expression is significantly increased in the connective tissue of facioscapulohumeral muscular dystrophy (a form of muscular dystrophy) [31].

While evidence is beginning to accumulate that FGFs will be involved in postnatal muscle maintenance and growth, their exact role has yet to be completely elucidated. In this manuscript, we examined FGFR1 expression during disuse-mediated skeletal muscle atrophy. We found FGFR1 to be expressed in muscle fibers within atrophying muscle, fibers which appeared to be resistant to atrophy. We also found that ectopic expression of FGFR1 within skeletal muscle fibers inhibited disuse mediated muscle atrophy. In muscles of suspended animals, FGFR1 overexpression stimulated protein synthesis. These results support the theory that FGFR1 plays a role in regulation of postnatal skeletal muscle maintenance, conserving a population of fibers which could be potentially used for mobility during times of stress. These results could offer potentially novel and efficient therapeutic options for attenuating muscle atrophy during aging, illness and spaceflight.

## Methods

### Hindlimb suspension

All experimental procedures involving mice were performed in accordance with guidelines set forth by the Purdue Animal Care and Use Committee. Hindlimb suspension (HS) was performed on mice using the tail cast model established by Morey et al. [37] and modified by Park and Schultz [38]. Mice were attached to a two-dimensional track system by their tails. This modification allows mice free access to food and water ad libitum, but prevents mice from placing a load on their hind limbs.

Twelve-week-old male mice (AKR/J, Jackson Labs, Bar Harbor, ME) were weighed and randomly assigned to either HS for 4 d (n = 12), for 7 d (n = 11), or kept in normal weight bearing/non-suspension (NS, n = 12) state. After suspension, body weights were recorded, mice were euthanized and muscle samples were collected. Gastrocnemius muscles were weighed, snap frozen in 2-methylbutane cooled in liquid nitrogen, and stored at -80°C for histological analysis. Frozen cross-sections 10 μm were cut at -23°C using a Microm HM500 Cryostat (Walldorf, Germany) and subbed to silane-coated slides.

### In situ hybridization

Sense and anti-sense RNA probes were generated using the full-length coding sequence for FGF-4 cloned into Bluescript (Stratagene, La Jolla, California) [24], forming a template for a MAXIScript in-vitro transcription (Ambion, Austin, TX) using a [^33^P] UTP label. FGFR1 probes were generated using reverse transcription protocols and PCR primers as described [40]. Briefly, cDNA templates for PCR were generated by reverse transcribing total RNA from mouse muscle. FGFR1 cDNA was amplified using the following forward and reverse primers: (AGAGACCAGCTGTGATGA) and (AATACGACTCACTATAGGGTGAGGAAGACAGAGTCCTCC), respectively, where the reverse primer contained the T7 promoter sequence. FGFR1 cDNA was electrophoresed and purified using the QIAquick Gel Extraction Kit (Qiagen, Valencia, CA). Purified FGFR1 cDNA was used as a template with the aforementioned MAXIScript in-vitro transcription (Ambion, Austin, TX) and [^33^P] UTP to generate riboprobes for in situ hybridization as outlined previously. Control probes were generated using either labeled sense probes or excess cold antisense RNA probes as described [41].

In situ hybridization was performed as described previously [40]. Briefly, sections were fixed with 4% (w/v) paraformaldehyde in 1 × phosphate buffered saline (PBS) for 20 min and rinsed twice for 5 min in 1 × PBS. Sections were overlaid with proteinase K solution (20 μg/ml proteinase K diluted in 50 mM Tris-HCl, 5 mM EDTA; pH 8.0) for 5 min and washed in 1 × PBS for 5 min. Sections were post-fixed as described previously in the 4% (w/v) paraformaldehyde solution. Slides were acetylated for 10 min using triethanolamine/acetic anhydride at pH 8.0. Sections were washed in 1 × PBS, dehydrated using absolute ethanol and air dried. Labeled probes were added to hybridization solution (4 × SSC, 1 × Denhardt's, 16 μg tRNA, 10% dextran sulfate, 50% formamide) at a final concentration of 50,000 cpm/μl. Sections were incubated overnight at 55°C. Following hybridization, sections were washed once for 15 min and twice for 30 min in wash solution (50% formamide in 2 × SSC at 65°C) and twice for 60 min in 35% formamide, 1 × SSC, 0.5 × PBS at 65°C. Three muscle sections from 10 animals from each treatment (control, 4 days hind limb suspension, and 7 days of hind limb suspension) were analyzed. Following hybridization, slides were subjected to a phosphor screen (Packard BioScience, Meriden, CT) overnight. Images were acquired using the Cyclone Storage Phosphor System (Packard BioScience, Meriden, CT), and analyzed using the Optiquant image analysis software provided with the system (Packard BioScience, Meriden, CT).

### Immunohistochemistry

Muscle cross-sections were blocked for 5 min at room temperature in a solution containing 0.3% horse serum (v/v) and 0.3% hydrogen peroxide (v/v) diluted in 1 × phosphate buffered saline (PBS). After blocking, sections were washed in 1 × PBS. The mouse-on-mouse immunoglobulin blocking solution (Vector M.O.M Immunodetection Kit; Vector Laboratories, Inc., Burlingame, CA) was used. Sections were blocked 1 h at room temperature in the mouse-on-mouse blocking solution. Slides were rinsed in 1 × PBS and incubated 5 min with the mouse-on-mouse working solution. Excess solution was removed and sections were incubated for 30 min with a mouse monoclonal FGFR1 antibody (QED Bioscience, San Diego, CA) diluted 1:2000 in working solution, or anti α-Actinin (Sigma-Aldrich, St. Louis, MO) diluted 1:800. Slides were rinsed in 1 × PBS and incubated 10 min at room temperature with biotinylated anti-mouse IgG. Sections were rinsed in 1 × PBS and exposed to an avidin-biotin-horseradish peroxidase complex (ABComplex^® ^Elite Kit; Vector Laboratories, Inc. Burlingame, CA). Slides were rinsed with 1 × PBS and reacted with 3,3'-diaminobenzidine substrate. Slides were washed in 1 × PBS, dried and coverslipped. Slides stained by immunohistochemistry for analysis were of muscle sections from the middle region of the muscle. Digital images were captured with a Leaf Microlumina camera (Scitex, Tel-Aviv, Israel) mounted on an Olympus BX50 microscope (Olympus, Melville, NY). Sections stained with anti α-Actinin were scanned at 20 × magnification and used to measure cross-sectional area of muscle fibers. A total of 16 fields were scanned from both legs from each mouse. From each field 10 randomly selected fibers were outlined using Adobe Photoshop^© ^(Adobe Systems, Inc., San Jose, CA) to determine cross-sectional area using the histogram function. A total of 800 myofibers were examined in the gastrocnemius from mice which had undergone 7 days of hindlimb suspension, while 960 fibers the gastrocnemius were examined from mice which had undergone 4 days of hindlimb suspension, and from control mice.

### DNA constructs

A full length cDNA encoding FGFR1 cloned into the MIRB expression vector was kindly provided by D.M. Ornitz (Washington University, St. Louis, MO). Plasmid DNA containing the full coding region of FGF-4 cloned into Bluescript (Stratagene, La Jolla, California) was provided by Lee Niswander (University of Colorado, Denver, CO). Control pcDNA 3.1 (+) plasmid DNA was purchased from Invitrogen (catalog no. V790-20, Carlsbad, CA). Cytomegalovirus (CMV)-Lac-Z encoding β-galatosidase was provided by B.B. Olwin (University of Colorado, Boulder, CO). Renilla luciferase vector containing the SV40 promoter region (pRL-SV40) was purchased from Promega (catalog no. E2231, Madison, WI). Plasmid DNA encoding for Runx2/Osf2 under the control of a cytomegalovirus promoter (pCMV-Osf2) and mouse osteocalcin gene 2 (mOG2) promoter fused to a luciferase reporter (pII 1.5 Luc) were gifts from R.T. Franceschi (University of Michigan, Ann Arbor, MI). Plasmid DNA encoding an ubiquitin-tagged luciferase (Ub-Fl) was a gift from D. Piwnica-Worms (Washington University, St. Louis). All DNA plasmids for animal injections were purified using Endo Free Plasmid Maxi Kits from Qiagen (catalog no. 12362, Valencia, CA).

### In-Vivo plasmid DNA transfection

Plasmid DNA was electroporated into skeletal muscle fibers of twelve-week-old ND4 male mice (Harlan, Indianapolis, IN) as described previously [42]. Mice were anesthetized using an intraperitoneal injection cocktail containing ketamine (9 mg/ml) and xylazine (1 mg/ml) in 0.9% saline at 0.01 ml/gram body weight. Hind limbs were shaved and the gastrocnemius and soleus muscles were injected with 50 μl of a 0.9% saline solution containing either 30 μg FGFR1 plasmid DNA and β-galactosidase (20 μg), or control plasmid (30 μg) and β-galactosidase (20 μg) in the contralateral limb through the skin. After DNA injection, electrotransfer gel was applied to each limb, and two plate electrodes connected to an ECM 830 electrical pulse stimulator (BTX^® ^inc., Holliston, MA) were positioned on the lateral and medial aspects of the leg adjacent to the muscle. Limbs were pulsed 8 times at 200 V/cm, 20 msec/pulse with a delay of 1 sec between pulses.

Forty-eight hours after electroporation, mice were randomly assigned to either HS for 7 d (n = 6), HS for 14 d (n = 6), or a reloaded treatment (n = 6). For the reloaded treatment, mice were suspended for 7 d, and then allowed to recover in a non-suspended, weight bearing status for 7 d. Following treatments, mice were euthanized and muscle samples were collected and frozen as described previously.

Gastrocnemius muscles were sectioned and stained for β-galactosidase [43]. Briefly, sections were fixed in 0.5% gluteraldehyde for 5 min at room temperature and rinsed in 1 × PBS. Sections were incubated in the dark overnight at 37°C in a solution containing 1 mg/ml X-Gal (5-bromo-4-chloro-3-indolyl-β D-galactopyranosidase; BP1615-1, Fisher Scientific, Fair Lawn, NJ), 10 mM K-ferricyanide, 10 mM K-ferrocyanide and 0.2 mM MgCl_2_, diluted in 1 × PBS. After color development, sections were rinsed in 1 × PBS and coverslipped. Images were captured with Scion Image for Windows Beta 4.0.2 (Scion Corporation, Frederick, MD) using a Nikon DN100 digital camera (Nikon Corporation, Tokyo, Japan) mounted on a Nikon Labphot light microscope (Nikon Corporation, Tokyo, Japan). Cross-sectional area of at least thirty β-galactosidase positive myofibers/per section/per animal were determined using Adobe Photoshop (Adobe Systems, Inc., San Jose, CA). Ten sections/animals were analyzed. This experiment was performed three times.

### Downstream detection of FGF signaling

Gastrocnemius and soleus muscles of mice were electroporated as described with 50 μl of a 0.9% saline solution containing 30 μg of plasmid DNA encoding FGFR1, a Runx2 expression plasmid (20 μg), a mOG2-luciferase reporter gene (20 μg) and CMV-renilla luciferase (10 μg). Contralateral control limbs received 30 μg of control plasmid DNA, Runx2 expression plasmid (20 μg), mOG2-luciferase reporter gene (20 μg) and renilla luciferase (20 μg). Five days after transfection mice were euthanized, and gastrocnemius muscle samples were frozen in liquid nitrogen. Muscle samples were powdered in a mortar and pestle cooled in liquid nitrogen and homogenized in passive lysis buffer (PLB) (catalog no. E1941, Promega, Madison, WI) containing leupeptin (2.1 μM/L), aprotinin, (0.15 μM/L), and phenylmethylsulfonyl fluoride (10 μM/L). Luciferase activity in each muscle sample was determined using the Dual-Luciferase^® ^Reporter Assay System (catalog no. E1960, Promega, Madison, WI), measured on a TD-20/20 luminometer (Turner Designs, Sunnyvale, CA). Luciferase activity of mOG2 was normalized to renilla luciferase activity in each sample to correct for transfection efficiency.

### Protein synthesis

ND4 male mice (Harlan, Indianapolis, IN) were electroporated into the gastrocnemius/soleus muscle, as described above, with 100 μg FGFR1 plasmid DNA. The contralateral muscle then received 100 μg of control plasmid DNA. Mice were randomly assigned to either HS treatment (n = 9) for 10 d, or normal weight bearing treatment for 10 d (n = 10). DNA transfections and HS were performed as previously outlined. To determine protein synthesis, a modification the flooding dose method described [44] was performed. Briefly, 250 μCi L-[4-^3^H] phenylalanine (catalog no. TRK204, Amersham, Piscataway, NJ) per mouse and 150 μmol unlabeled phenylalanine/100 g body weight was administered intraperitoneally. Thirty min after dosing, mice were euthanized, hindlimbs were skinned, and placed on ice for dissection. Gastrocnemius and soleus muscles were removed, snap frozen in liquid nitrogen and stored at -80°C for later analysis.

Frozen samples were powdered using a mortar and pestle cooled in liquid nitrogen and homogenized in PLB as described previously. Protein concentration of the homogenate was determined using the bicinchoninic acid protein assay (catalog no. 23225, Pierce, Rockford, IL). A volume containing 200 μg of total protein was precipitated in one quarter volume 0.2 M perchloric acid (PCA) and pelleted. Pellets were washed twice with 0.2 M PCA and solubilized using 0.5 ml of 0.5 M NaOH. Solubilized pellets were added to 10 ml EcoLite™(+) scintillation fluid (catalog no. 882475, ICN Biomedicals, Inc., Irvine, CA) and counted using liquid scintillation (Model B1600, Packard BioScience, Meriden, CT). This experiment was performed three times.

### Proteasome activity

Proteasome activity was assayed using the ubiquitin-luciferase reporter as described by Luker et al [45]. Gastrocnemius and soleus muscles of 12-week-old ND4 male mice (Harlan, Indianapolis, IN) were electroporated with a 0.9% saline solution containing either 30 μg FGFR1, Ub-Fl (20 μg), and renilla luciferase (10 μg), or control (30 μg), Ub-Fl (20 μg), and renilla luciferase (10 μg) plasmid DNAs. The ubiquinated luciferase construct synthesizes a ubiquitin-luciferase fusion protein that is degraded by, and an indicator or activity of, the ATP-depended proteosome. Mice were randomly assigned to either HS treatment (n = 5) for 10 d, or remained in weight bearing treatment (n = 4) for 10 d. At the conclusion of the treatment period, mice were euthanized, muscles were collected and processed as described previously for determining firefly and renilla luciferase activity.

### Statistical analysis

To test the effect of FGFR1 treatment and hindlimb unloading on muscle fiber area (CSA), tritium incorporation, and proteasome activity, a general linear model of SAS was used to perform the analysis of variance. A mixed model with mouse as a blocking factor for repeated measures analysis was performed on contralateral limbs as repeated measurements within each mouse. The data from the protein synthesis study showed a poisson distribution, so data were transformed (square root). Probability values less than 0.05 were considered statistically different.

## Results

### Muscle morphology

In comparison to the weight bearing controls, after 7 days of hindlimb suspension fiber CSA was significantly decreased in the gastrocnemius by an average of 311 μm^2 ^(control : 1688 μm^2^; 4 days of hind limb suspension : 1454 μm^2^; 7 days of hind limb suspension : 1277 μm^2^; SEM = 52 μm^2^; all means significantly different p < .05).

### In-situ hybridization

**Figure 1:**
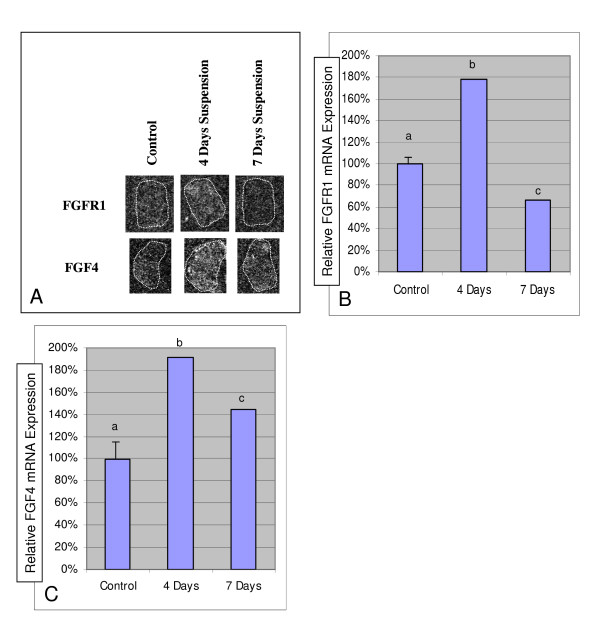
**FGFR1 and FGF4 mRNAs are up-regulated in muscle undergoing disuse atrophy**. FGFR1 and FGF4 mRNAs were analyzed in sections of gastrocnemius muscle obtained from control mice or mice suspended for 4 or 7 days by *in situ *hybridization. In comparison to controls, FGFR1 and FGF4 mRNA signal, as depicted by white silver grains in dark field images, were upregulated in gastrocnemius muscles suspended for 4 days (A). Direct dpm analyses were obtained using the Cyclone Storage Phosphor System, and FGFR1 and FGF4 mRNA levels were found to be significantly greater in muscles suspended for 4 days than control muscle or muscle suspended for 7 days. Dotted lines represent the outline of the gastrocnemius muscles. SEM indicated over control; means with different superscripts differ p < .05; n = 10 for each mean.

Quantitative in-situ hybridization of the gastrocnemius using probes for FGFR1 and FGF-4 demonstrated detectable levels of growth factor expression in control animals. Hind limb suspension significantly altered the expression of FGFR1 and FGF4 after 4 and 7 days when compared with weight bearing controls. Following 4 days of disuse FGFR1 mRNA expression was significantly increased, with expression levels at 178% of controls (p < 0.05). After 7 days of disuse FGFR1 mRNA expression was significantly decreased, with expression at only 66% of controls (p < 0.05; Figure 1.A,B). FGF-4 mRNA levels were significantly elevated after 4 days of hind limb suspension at 192% of control (p < 0.01; Figure 1.A,C). After 7 days of disuse FGF-4 expression was still significantly elevated when compared to controls (145% of control; p < 0.01; Figure 1.A,C), but was significantly lower than expression levels noted after 4 days of disuse (p < 0.01; Figure 1.A,C). Control sense ribroprobes did not yield significant hybridization signal (data not shown).

### FGFR1 immunohistochemistry

**Figure 2:**
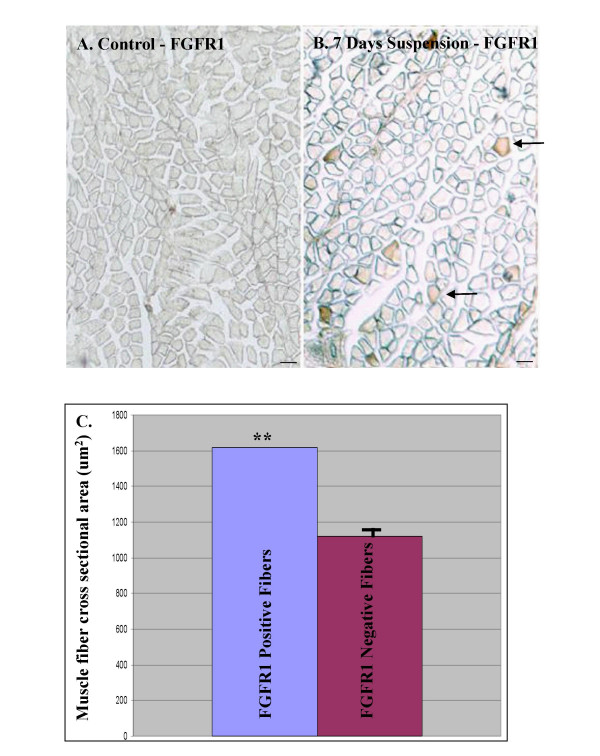
**FGFR1 protein is up-regulated in muscle fibers during disuse atrophy**. FGFR1 was immunolocalized in sections of gastrocnemius muscle obtained from control mice or mice suspended for 7 days. In comparison to the control gastrocnemius (***A***), FGFR1 protein, as depicted by the brown precipitate (black arrows), was increased in atrophying muscle (***B***). In sections of the gastrocnemius isolated from suspended mice, the area of the FGFR1-positive muscle fibers was significantly greater than the FGFR1 negative fibers (***C***). The area of the FGFR1 positive myofibers in the atrophying muscle was the same as the area of control myofibers (1688 mm2). This data suggests that FGFR1 acts to inhibit fiber atrophy associated with disuse. ** indicates p < .01; SEM indicated; bar represents 50 mm.

A population of fibers immunostained strongly positive for FGFR1 in gastrocnemius muscles from mice following 7 days of disuse atrophy (Figure 2.A,B). This localized increase in FGFR1 immunoreactivity was not detected in control muscle. The average CSA of fibers expressing increased levels of FGFR1 protein in atrophied muscle was significantly larger than fibers not demonstrating this increase (in atrophied muscle) by 522 μm^2 ^(p < 0.01; Figure 2.C). The average diameter of the FGFR1 expressing fibers in atrophying muscle (1615 μm^2^) was similar to that of the weight bearing controls (1688 μm^2^).

### Overexpression of FGFR1 in muscle blunts muscle atrophy

**Figure 3:**
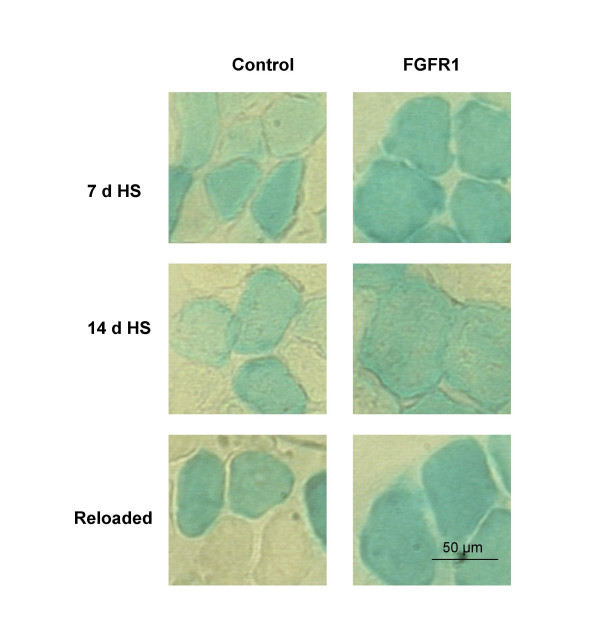
**Ectopic expression of FGFR1 inhibits atrophy**. β-galactosidase expression in muscle fibers from gastrocnemius muscles of mice co-transfected with a β-galactosidase expression vector and either a control (control) or FGFR1 expression plasmid DNA (FGFR1). Rows represent fibers from muscles of mice suspended for 7 d (7 d HS), 14 d (14 d HS), or reloaded for 7 d after 7 d HS (reloaded). The bar in the bottom right figure represents 50 μm.

**Figure 4:**
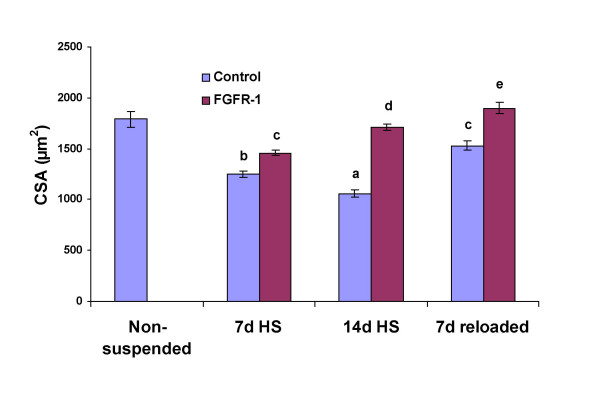
**Overexpression of FGFR1 inhibits muscle atrophy**. Cross-sectional area (CSA) of fibers from gastrocnemius muscles of mice transfected with either control (control) or FGFR1 expression plasmid DNA (FGFR1), subjected to hindlimb suspension for 7 d (7 d HS), 14 d (14 d HS), reloaded for 7 d after 7 d of hindlimb suspension (7 d reloaded), or non-suspended controls (NS). Means (±SEM) bearing different letters differ significantly (P < 0.05).

To determine if the elevated FGFR1 was playing a direct role in maintaining muscle fiber size during disuse atrophy, muscle fibers were electroporated with an FGFR1 expression plasmid and subjected to HS. After 7 d HS, fibers expressing electroporated FGFR1 were 17% larger (*P *< 0.05) than those fibers from the contralateral control limbs expressing a control plasmid. Similarly, fibers expressing electroporated FGFR1 in mice suspended 14 d were 62% larger (*P *< 0.01) than fibers from the contralateral control limbs expressing a control plasmid. In reloaded animals, muscle fibers expressing electroporated FGFR1 were 24% larger (*P *< 0.01) than contralateral control muscle fibers expressing control plasmid (Figures 3 and 4). These results demonstrate that FGFR1 plays an active role in inhibiting the loss in muscle fiber diameter that occurs during unloading. No significant difference was noted in the CSA of fibers transfected with FGFR1 and LacZ when compared with fibers transfected with only LacZ in the muscles of weight bearing mice (LacZ expressing fibers: 1623 μm; FGFR1/LacZ expressing fibers: 1615 μm; SEM = 323).

### FGFR1 in muscle regulates protein synthesis and degradation

**Figure 5:**
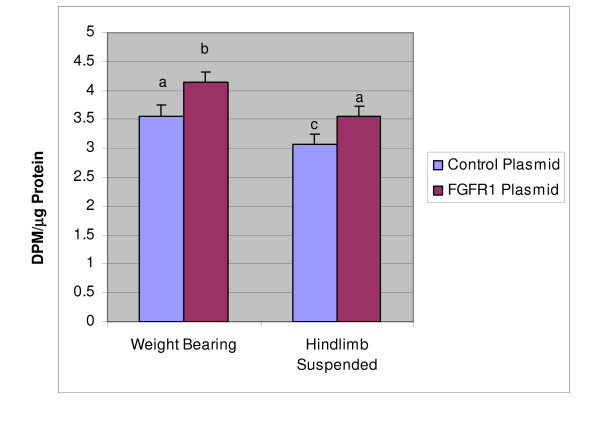
**Overexpression of skeletal muscle with FGFR1 increases protein synthesis**. Incorporation of L-[4-^3^H] phenylalanine in gastrocnemius and soleus muscles of weight bearing or hindlimb suspended mice transfected with control (control) or FGFR1 expression plasmid DNA (FGFR1). Values represent back transformation of means (+SE/-SE) generated using a square root transformation. Values bearing different letters differ significantly (*P *< 0.05).

To determine the effects of FGFR1 on protein turnover, protein synthesis and protein degradation were evaluated in FGFR1 electroporated muscles. Gastrocnemius/soleus muscles from mice subjected to HS for 10 d incorporated less (*P *< 0.05) tritium than those of unsuspended mice (Figure 5). These results confirm previous studies that demonstrate a reduction of protein synthesis that occurs during hind limb suspension [46]. In both weight bearing and suspended gastrocnemius/soleus muscles electroporated with FGFR1, there was an average 16% greater (*P *< 0.05) tritium incorporated than in contralateral muscles electroporated with control plasmid (Figure 5). These results show that ectopic expression of FGFR1 stimulates protein synthesis in skeletal muscle fibers in vivo.

**Figure 6:**
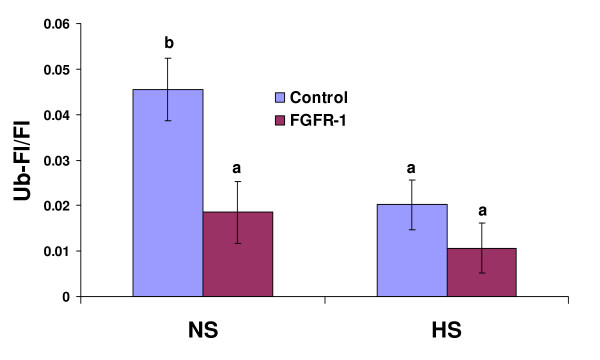
**Proteasome activity is unaffected by overexpression of FGFR1 in muscle subjected to hindlimb suspension (HS)**. Luminescence of extracts from the gastrocnemius and soleus muscles of mice transfected with either control (control) or FGFR1 expression plasmid DNA (FGFR1), subjected to either hindlimb suspension (HS), or non-suspended controls (NS). Data are expressed as luciferase dependent light units of Ub-Fl normalized to renilla luciferase dependent light units. Means (±SEM) bearing different letters differ significantly (P < 0.05).

The 26S proteasome represents a key component in ubiquitin-mediated proteolysis, so activity of the proteasome was used to determine the regulation of proteolysis by FGFR1. The proteasome reporter plasmid encodes a poly-ubiquitinated sequence linked to luciferase [45]. Higher luciferase activity indicates lower proteasomal activity, and thus interpreted as lower proteolysis. Proteasome activity was significantly (*P *< 0.01) increased 2.3 fold in muscles from control, suspended muscle as compared to muscles isolated from weight bearing control mice. This data is in agreement with previous studies showing proteolysis is increased in muscle undergoing hind limb suspension induced-atrophy [45]. Proteasome activity of muscles from suspended mice electroporated with FGFR1 was not significantly different from contra-lateral muscles electroporated with control plasmid. In muscle from weight-bearing control mice electroporated with FGFR1, there was a 2.5 fold increase (*P *< 0.01) in proteosome activity (Figure 6) as compared to the contralateral muscle electroporated with control plasmid.

### Downstream detection of FGF signaling in muscle

**Figure 7:**
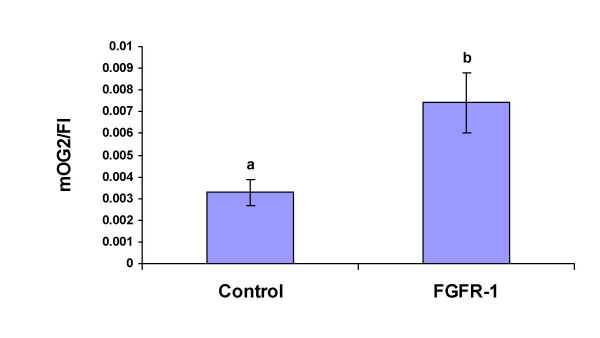
**Downstream detection of activated FGF signaling**. Activity of mouse osteocalcin gene 2 promoter (mOG2)-luciferase reporter gene in gastrocnemius and soleus muscles of mice transfected with either control (control) or FGFR1 expression plasmid DNA (FGFR1). Data are expressed as luciferase dependent light units normalized to renilla luciferase dependent light units (mOG2/Fl). Means (±SEM) bearing different letters differ significantly (*P *< 0.01).

As described previously [9], we sometimes have difficulty using immunohistochemistry to display ectopic plasmid protein expression. Although we used muscles that showed no overt necrosis and mineralization in response to electroporation, there still seemed to be an insult to the muscle that appeared as a strong affinity for the secondary antibody during immunohistochemistry. Even on sections from muscles 30 days post-electroporation, there was a strong background of secondary antibody immunostaining, that we could not block with MOM kits (Mouse On Mouse, Vector Laboratories) or excess quenching with unlabeled secondary antibodies. Therefore, to verify FGF signaling was stimulated by overexpression of FGFR1, a reporter assay using plasmid DNAs for Runx2 and mOG2-luciferase as described by Xiao et al. [47] was utilized. FGF signaling is required for Runx2 phosphorylation, which is required for activation of OG2 transcription. Increased mOG2 reporter gene activity is indicative of increased FGF-signaling. Gastrocnemius and soleus muscles electroporated with FGFR1 plasmid DNA exhibited a two-fold increase (P < 0.05) in OG2 luciferase activity as compared to control muscles electroporated with control plasmid (Figure 7). These results verify that FGF-signaling was stimulated by ectopic expression of FGFR1.

## Discussion

FGFs are demonstrated regulators embryonic skeletal muscle growth and repair. However, their importance during adult muscle maintenance in vivo has not been examined. In this manuscript, we found FGFR1 to be synthesized in a population of muscle fibers within atrophying muscle which appeared to be resistant to reduced load. We also found that ectopic expression of FGFR1 within skeletal muscle fibers in vivo inhibited disuse mediated muscle atrophy through a mechanism that included stimulation of protein synthesis. In muscle fibers bearing a load, we found ectopic FGFR1 stimulates protein synthesis and degradation.

FGFRs have been implicated in regulation of myoblast proliferation and differentiation [48], but an FGFR role in fiber maintenance post-differentiation has not been widely reported. The position of FGFR1 as a cell surface receptor places it in a position where it may affect many cellular processes, as well as the response of an individual fiber to the surrounding micro-environment. The elevated expression noted in individual myofibers could indicate that increased FGFR1 confers some form of protection from atrophy by increasing the sensitivity of the myofiber to whatever soluble growth factors are present. Our work demonstrated that there was a concurrent increase in expression of at least one ligand in atrophying muscle, FGF4. Significant increases in FGF4 expression have been previously noted in a model of skeletal muscle hypertrophy [49], but have not been previously reported in any models of disuse or atrophy.

Our results would indicate FGFR1 has a role in maintaining postnatal muscle fiber size and strength in a specific population of myofibers during periods of disuse. When a muscle undergoes atrophy during reduced load such as the hind limb suspension model, proteins are degraded into amino acids via the ATP-dependent proteosome [7]. These amino acids can then converted into glucose by the liver and then utilized by the brain. It would be by this mechanism that a body keeps the brain functioning at a time of stress at the expense of other immediately "less important" organs such as the skeletal muscle. However, for optimal survival at some point post-stress it would still be important to have some ability to move, and not have all muscle fibers significantly depleted. Our work suggests a model where FGFR1 would conserve a population of muscle fibers during an atrophic phase for an unknown period of time in order to allow some ability of post-stress escape or movement. This sparing effect seemed to be specific for FGFR1, as RT-PCR analysis demonstrated no change in FGFR4 expression in response to hind limb suspension and FGFR2 and 3 are not expressed in adult skeletal muscle (data not shown). In cultured muscle, FGFR1 expression is typically associated with the proliferative state of muscle, while FGFR4 expression is correlated with the differentiated myofiber [50]. However, in muscle in vivo the expression patterns of FGFR1 and FGFR4 are not as clear cut. Satellite cells isolated from the Extensor digitorum longus and Soleus muscles differ in the expression of FGF receptors. FGFR1 and R4 mRNA were strongly expressed in proliferating cultures whereas in differentiating cultures, only FGFR1 was present in EDL satellite cells while FGFR4 was also still expressed in Soleus cells. In human muscle FGFR1 immunoreactivity has been demonstrated in localized areas of myofibers [51]. These observations, in addition to our work, add support to the theory that FGFR1 regulation of a muscle fiber is much more complex than simply regulating embryonic myoblast differentiation.

**Figure 8:**
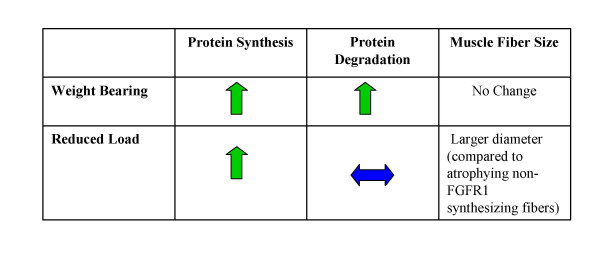
**Proposed role of FGFR1 in adult skeletal muscle**. Increased production of FGFR1 in a weight bearing muscle fiber would result in an increase in protein synthesis and degradation, which would counteract each other and result in no observable increase in muscle fiber size. However, in a fiber undergoing atrophy, increased FGFR1 would not elevate the significant amount of proteolysis that would already be occurring in an atrophying fiber. However, FGFR1 would still elevate protein synthesis in the atrophic fiber, thereby maintaining fiber size and strength.

We did not observe a localized accumulation of FGFR1 in weight-bearing muscle as found in atrophying muscle. However, we still found detectable mRNA for FGFR1. This would suggest that FGFR1 can be expressed in and exert a biological effect weight-bearing differentiated myofibers, as it does in unloaded muscle fibers. The actual biological effect of FGFR1 in weight bearing muscle would be less clear. In our studies the ability of FGFR1 to regulate fiber size only occurred during disuse atrophy, as ectopic expression of FGFR1 in weighted muscle fibers stimulated protein synthesis and degradation, but had no significant phenotypic effect (See Figure 8). Even though there is no direct effect on fiber size, it is possible that FGFR1 is stimulating or involved in a fiber type conversion or reconstruction. As mentioned above, FGFR1 expression is more prevalent in fast muscles such as the EDL. This could account for the increased degradation as the "old type" proteins were being degraded in preparation for the increased synthesis of the "new type" proteins/enzymes. The effects of FGF signaling on fiber type conversion are currently being examined.

It is known that FGFR signals via the Ras-Raf-MEK-MAPK pathways. Activation of this pathway via acute up-regulation of FGF-signaling promoted protein degradation in differentiated muscle fibers from *Caenorhabditis elegans *[52]. Our results confirm that this protein turnover regulating property of FGFR is conserved in mammalian muscle fibers and demonstrates a link between FGF signaling and the ATP-dependent proteasome pathway. The fact that FGFR1 activation of proteolyses in weight-bearing muscle contrasts from its function in unloaded muscle might simply be due to the fact that proteolysis is already activated in unloaded muscle and overexpression of FGFR1 can not exert any more of an effect on that process.

In summary, our results support a theory that FGFs play crucial roles in regulation of postnatal skeletal muscle maintenance. As the mechanisms regulating skeletal muscle growth, atrophy, and hypertrophy are more clearly elucidated, effective treatments for conditions resulting in a loss of skeletal muscle function will be further developed. The effects of FGFR1 noted in this study make this growth factor receptor a strong candidate for future study of its therapeutic or preventative potential.

## Competing interests

The author(s) declare that they have no competing interests.

## Authors' contributions

JE and AO carried out the animal and molecular studies. JE carried out the protein synthesis and degradation assays. JE, AO, GB, DG and KH participated in the design of the study and performed the statistical analysis. JE, AO, GB, DG and KH all conceived the aspects of this study, and participated in its design and coordination. All authors read and approved the final manuscript.

## Pre-publication history

The pre-publication history for this paper can be accessed here:
